# A Generic Quantitative Risk Assessment Framework for the Entry of Bat-Borne Zoonotic Viruses into the European Union

**DOI:** 10.1371/journal.pone.0165383

**Published:** 2016-10-27

**Authors:** Robin R. L. Simons, Verity Horigan, Paul Gale, Rowena D. Kosmider, Andrew C. Breed, Emma L. Snary

**Affiliations:** Animal and Plant Health Agency (APHA), Department of Epidemiological Sciences, New Haw, Addlestone, Surrey, KT15 3NB, United Kingdom; University of Pretoria, SOUTH AFRICA

## Abstract

Bat-borne viruses have been linked to a number of zoonotic diseases; in 2014 there have been human cases of Nipah virus (NiV) in Bangladesh and Ebola virus in West and Central Africa. Here we describe a model designed to provide initial quantitative predictions of the risk of entry of such viruses to European Union (EU) Member States (MSs) through four routes: human travel, legal trade (e.g. fruit and animal products), live animal movements and illegal importation of bushmeat. The model utilises available datasets to assess the movement via these routes between individual countries of the world and EU MSs. These data are combined with virus specific data to assess the relative risk of entry between EU MSs. As a case study, the model was parameterised for NiV. Scenario analyses showed that the selection of exporting countries with NiV and potentially contaminated trade products were essential to the accuracy of all model outputs. Uncertainty analyses of other model parameters identified that the model expected number of years to an introduction event within the EU was highly susceptible to the prevalence of NiV in bats. The relative rankings of the MSs and routes, however, were more robust. The UK, the Netherlands and Germany were consistently the most likely points of entry and the ranking of most MSs varied by no more than three places (maximum variation five places). Legal trade was consistently the most likely route of entry, only falling below human travel when the estimate of the prevalence of NiV in bats was particularly low. Any model-based calculation is dependent on the data available to feed into the model and there are distinct gaps in our knowledge, particularly in regard to various pathogen/virus as well as host/bat characteristics. However, the strengths of this model lie in the provision of relative comparisons of risk among routes and MSs. The potential for expansion of the model to include other routes and viruses and the possibility of rapid parameterisation demonstrates its potential for use in an outbreak situation.

## 2 Introduction

In the current era there are many factors which connect disparate parts of the world, from tourism to immigration and legal trade of goods to illegal trade of wild animals. However, along with the inarguable benefits of such globalisation comes risk to human health. One of the biggest risks of an interconnected world is the potential spread of zoonotic diseases. In recent times, we have seen geographical spread of cases of viruses such as Avian Influenza, Middle East Respiratory Syndrome coronavirus (MERS-CoV) and Ebola virus (EBOV) at unprecedented distances from the initial source of the outbreak. This raises the concern that other viruses that are currently restricted to more localised geographic areas, such as Nipah Virus (NiV), may spread to many other countries. If entry of the virus occurs, there is the possibility of exposure and transmission to humans, wildlife and/or livestock. As an example, an airline passenger infected with EBOV entered Nigeria from Liberia and infected nine medical staff and an airport official [1]. It is likely that such events will be sporadic and that, within the EU, there will be rapid implementation of control measures to contain them. However, if the initial control measures were to be ineffective, free trade and movement between European Union (EU) Member States (MSs) could increase the risk that disease arising within one MS could quickly spread within the EU. Spread within the EU will of course depend on a number of other factors, such as whether the virus can get established, but if unprepared, the consequences of virus entry could potentially be severe leading to an epidemic, or even pandemic. Such consequences have been witnessed in West Africa where over 20,000 suspected cases and 7,800 deaths have been reported due to the 2014 EBOV outbreak [2], which has been suggested originated from only one human index case involving spill-over from a wildlife reservoir [3].

Bats are a known reservoir for a number of zoonotic viruses including NiV, and Marburg virus and are also linked to other zoonotic diseases such as EBOV, and MERS-CoV [4–8]. Bats infected with such viruses seldom display clinical signs but may shed virus in urine, faeces or saliva [6, 9]. As such, they are a likely vector for both direct and indirect transmission to humans and other animals. There have been cases of NiV in Bangladesh most years since it was first documented in 2001; in 2014 there were 30 human cases including 18 deaths [10]. The main risk factor for initial human infection with the Bangladesh strain of NiV is thought to be consumption of date palm sap contaminated by bats [11], but more than half of the identified cases have resulted from person-to-person transmission [12]. Looking ahead, particularly with current anthropogenic activities (e.g. deforestation, hunting of wildlife for bushmeat), it is possible that there will be further spill-overs of viruses from bat populations to local wildlife and human populations in areas where there have previously been limited interactions [13]. This coupled with our intricately connected world through trade and travel provides new opportunities for wide-spread transmission of the viruses upon the initial spill-over event.

While there have been a number of recent rapid risk assessments in response to the MERS-CoV and Ebola outbreaks [14–17], including assessments of the risk of individual routes [18, 19], there are few in-depth risk assessments, either qualitative or quantitative, considering the risk of introduction of bat-borne zoonotic viruses into naïve human populations at a regional level. Of note is a qualitative risk assessment of the introduction of henipaviruses into the United Kingdom (UK), which identified a number of different pathways by which these viruses could enter the UK and highlighted high levels of uncertainty due to data gaps [20].

This paper aims to develop a generic model framework to quantitatively assess the risk of introduction of bat-borne zoonotic viruses into the EU. The Bangladesh strain of NiV, an emerging paramyxovirus, has been selected as a case study for demonstrating the model function and outputs, due to its pandemic potential and link to humans through a non-animal food product (i.e. date palm sap). However, the model framework has been designed such that it can be readily parameterised for other viruses (e.g. EBOV and MERS-CoV).

In this paper we first present the generic model framework, before describing the NiV specific parameterisation. Model results for the NiV parameterisation are presented, including scenario analyses, followed by a discussion on the issues surrounding development and parameterisation of a generic model framework. The overall aim of this work was to develop a model that could be parameterised and run relatively quickly and easily, in order to provide a quantitative assessment of risk that could be of benefit for researchers and policy makers in the early stages of a novel virus outbreak. While this aim has been achieved, there is a reasonable level of uncertainty in model outputs, due to the need for quality data to be available for all countries and the data gaps surrounding virus specific parameters such as prevalence and viral loads in animal species.

## 3 Materials & Methods

### 3.1 Overview

The model framework follows the World Organisation for Animal Health (OIE) code for import risk analysis [21]. Under traditional OIE guidelines, there are three components of risk assessment: entry assessment, exposure assessment and consequence assessment. This framework only considers the entry assessment, stopping at the point at which the virus is introduced into the EU, which we define to be prior to checks at customs/border inspection posts. Thus, any infected connecting passengers (i.e. passengers who do not leave the airport before boarding another flight out of the country) and/or contaminated products stopped or seized at EU customs or border inspection posts are considered to have constituted an introduction event. The model does not consider the potential exposure of humans, livestock or wildlife to the virus and the subsequent consequences given incursion.

The model is coded in the R software package. Due to the wide scope of the model, the need for data to be available for all countries and the desire for the model to be used at short notice to address questions in an outbreak situation, the baseline model is deterministic. Uncertainty and variability are considered in a series of analyses using alternative parameter values, with some analyses incorporating stochastic variability of specific parameters. The outputs of these analyses should be considered alongside the baseline model results as plausible alternatives.

### 3.2 Output

The main output of the baseline model is an estimate of the annual probability of at least one introduction event into each EU MS, *j*, *P*_*V*_*(j)*. This is derived by combining the probability of at least one introduction event from each of the routes included in the model, to produce an overall probability for each MS

$$P_{v}(j)=1-\prod_{r=1}^{R}\left(1-P_{r}(j)\right),$$ (1)

where *R* is the total number of routes considered for the virus and *P*_*r*_*(j)* is the probability of at least one introduction event via route *r* to MS *j* per year. The average number of years to an introduction event was calculated,

*Y*_*V*_*(j)* = 1/*P*_*V*_*(j)*, as well as ranking the risks between all 27 EU MSs, *Z*_*V*_*(j) = {1*:*27}*, to give an indication of where in the EU an introduction event could be more likely.

### 3.3 Routes included in the baseline model

Risk assessments for the importation of zoonotic viruses such as NiV and classical rabies into specific MSs have been undertaken previously [20, 22, 23]. In addition to this, workshops have been undertaken within specific EU MSs to investigate the most likely pathways for importation of exotic animal diseases [24–26]. Using this information, in conjunction with the risk factors identified as important in a previous review [27], the main routes included in the baseline model were human travel, *P*_*H*_*(j)*, legal trade of ‘at risk’ products (e.g. non-animal foodstuffs such as fruit products and products of animal origin), *P*_*G*_*(j)*, legal movement of susceptible animals (e.g. livestock, companion animals and those destined for scientific research or zoos), *P*_*A*_*(j)*, and illegal bushmeat trade, *P*_*B*_*(j)*. As Eq 1 is multiplicative with respect to the routes, the choice of routes can be amended for different viruses; e.g. inclusion of other routes, such as movement of bats, both accidental (e.g. stowaway in a boat) and through natural migration, that were not considered in the baseline model as previous research considered them to be of lower priority [27].

### 3.4 Model Framework

**Fig 1:**
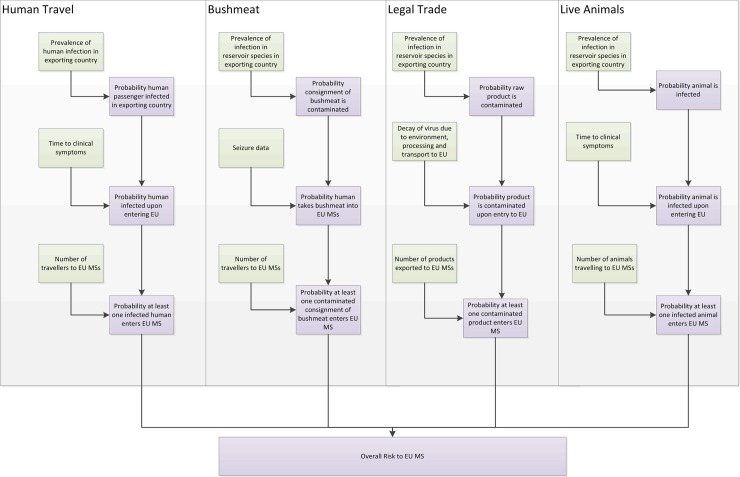
Overview of model framework, showing the important events in each route up to the point of entry to the EU. Green boxes highlight model parameters/data inputs and purple boxes highlight model estimates.

The risk assessment model is initiated by determining the ‘exporting countries’, i.e. countries where virus infection is strongly suspected to be circulating in bats, humans, or other animal species. For each route, the risk of an introduction event (i.e. importation of at least one infected/contaminated product per year) from each selected exporting country to each EU MS was assessed, by calculating the annual probability that at least one product (i.e. human, animal, product of animal origin or foodstuffs) is infected/contaminated upon entry into the MS. This estimate takes into account factors such as the probability an individual unit is infected (or contaminated) in the exporting country, the survival of the virus over the duration of the journey, whether the animal/human displays clinical signs and the annual volume of products being imported. Fig 1 provides an overview of the generic model framework.

### 3.5 Model Equations

#### Human travel

Human travel, within an ever-expanding air network, has become an increasingly important pathway for the rapid introduction and proliferation of infectious diseases in recent years. This was demonstrated by the SARS-CoV outbreak in 2003, where within weeks the disease infected over 8,000 individuals in 26 countries across 5 continents [28], and to a lesser extent the 2014 West Africa EBOV outbreak where infection spread from Liberia to Nigeria via a passenger on a commercial airline [1].

Passengers travelling to MS *j*, will likely have been in the exporting country *k* for a variety of different reasons, e.g. a resident of MS *j* going on holiday, business or visiting friends or relatives (VFR), or a resident of exporting country *k* going to MS *j* for similar reasons. The risk of exposure to and infection with viruses may differ between these groups. For example, a major route of NiV-Bangladesh transmission to humans is through consumption of raw date palm sap [29, 30], a traditional practice in some rural Bangladesh communities, but one less likely to be practiced by people visiting on business. Additionally, visitors to exporting country *k* may only be there a short while and thus have a shorter time for exposure than local residents. Thus, to account for these differences, the baseline model differentiates by passenger type.

Let *N*_*H*_*(i*,*j*,*k)* be the total number of passengers of type *i* travelling from country *k* to MS *j* in one year and *p*_*Hin*_*(i*,*k)* be the probability of a passenger of type *i* being infected upon entering the EU, then the probability that at least one infected human enters MS *j*, *P*_*H*_*(j)*, is given by

$$P_{H}(j)=1-\prod_{k=1}^{K}\prod_{i=1}^{I}\;{\left(1-p_{Hin}(i,k)\right)}^{N_{H}(i,j,k)},$$ (2)

where *I* is the total number of passenger types and *K* is the total number of exporting countries considered in the model.

It is assumed that any individual exhibiting clinical symptoms prior to travelling will not travel as planned, either due to the severity of the symptoms or being prevented from boarding by the relevant authorities. It is possible for a passenger infected with a zoonotic virus to develop clinical signs rapidly during travel. However, it is assumed here that those passengers will continue to their final EU MS destination, thus constituting an introduction event. Therefore, the infected population that may travel are those individuals currently undiagnosed and incubating disease (i.e. sub-clinically infected). This population is estimated by multiplying the prevalence of infection in passenger type *i*, *θ(i*,*k)* by the incubation period of the virus, *T*_*IP*_*(k)*. Based on previous work [23], it is assumed that the incubation period follows a log normal distribution and new infected cases follow a Poisson process with rate *λ(i*,*k)*

$$\lambda (i,k)∼Gamma\left(\theta (i,k)\;*\;\frac{T_{IP}(k)}{365},1\right).$$ (3)

As the baseline model is deterministic, the mean of this distribution is used to describe the probability of a passenger of type *i* being infected upon entering the EU

$$p_{Hin}(i,k)=\bar{\lambda }(i,k)=\theta (i,k)\;*\;\frac{T_{IP}(k)}{365}.$$ (4)

To account for differences in prevalence between passenger types, the baseline prevalence of infection in the exporting country is weighted by the average duration of stay (days) in the exporting country, *T*_*DK*_*(i*,*k)*,

$$\theta (i,k)=\frac{n_{H\inf}(k)}{N_{pop}(k)}\;*\;Min\left(1,\frac{T_{DK}(i,k)}{365}\right),$$ (5)

where *n*_*Hinf*_*(k)* is the number of human infections per year in the exporting country and *N*_*pop*_*(k)* is the population of the exporting country. Note that if *T*_*DK*_*(k)* exceeds 365 days then the passenger is there for the whole year and so the risk is equal to the baseline human prevalence in country *k*.

#### Legal trade import

There are large trade volumes of various products into the EU, including from many countries where bat-borne zoonotic viruses are present. Some trade products, such as fruit, may have been contaminated with virus from wildlife reservoir species in the exporting country, thereby posing a risk to the EU if the virus survives the journey. The Europhyt database contains information on interceptions of harmful organisms in commodities imported into the EU [31]. A few interceptions in recent years have been due to the presence of viruses, but none were bat-borne viruses. The commodities intercepted generally have shown visible signs of contamination, which may not be the case with the viruses considered here, making detection less likely [31]. Additionally, products from susceptible animal species may also contain infectious virus depending on the severity and dissemination of the infection in the animal.

Let *N*_*G*_*(j*,*k*,*l*,*m)* be the number of products of type *l* from exporting country *k* to MS *j* via transport route *m* in one year, then the probability that at least one contaminated trade product enters MS *j* in a year, *P*_*G*_*(j)*, is given by

$$P_{G}(j)=1-\prod_{k=1}^{K}\prod_{l=1}^{L}\prod_{m=1}^{M}\;{\left(1-p_{Gin}(j,k,l,m)\right)}^{N_{G}(j,k,l,m)},$$ (6)

where *L* is the total number of products, *M* is the total number of transport routes and *p*_*Gin*_*(j*,*k*,*l*,*m)* is the probability a trade product is contaminated upon entering MS *j*.

To determine whether a product is contaminated on arrival to an EU MS the model considers the prevalence of contamination in the raw product, *p*_*Graw*_*(k)*, the initial concentration of virus on a raw product in the exporting country and any reduction in viral load between initial contamination of the raw product and arrival in the EU MS. Let *c*_*0*_*(x)* be the probability density function associated with the initial viral load on a contaminated raw product and *a(j*,*k*,*l*,*m)* be the initial concentration necessary for the product to still be considered contaminated upon arrival at the EU MS

$$\begin{array}{ccc} p_{Gin}(j,k,l,m) & = & p_{Graw}(k)P\left(x>a(j,k,l,m)\right)\\ & = & p_{Graw}(k)\left(1-P\left(x\leq a(j,k,l,m)\right)\right)\\ & = & p_{Graw}(k)\left(1-\int_{x=0}^{a(j,k,l,m)}c_{0}(x)dx\right) \end{array}.$$ (7)

The calculation of *a(j*,*k*,*l*,*m)* will vary depending on the data used to parameterise it, e.g. it could be a simple point value, or an estimate based on expected reductions in virus concentration at different stages of the trade chain. The baseline model is set up for possible reduction of virus on the product prior to harvesting, *C*_*env*_*(k*,*l*), during processing, *C*_*proc*_*(k*,*l)*, and during transport to the EU MS, *C*_*trans*_*(j*,*k*,*l*,*m)*. The reduction at processing is a simple log reduction based on the type of processing undertaken, while the reductions at harvesting and during transport are calculated based on the half-life of the virus during that stage (*C*_*HLenv*_*(k*,*l)* and *C*_*HLtrans*_*(k*,*l)* respectively) and the duration of time spent in the stage (*T*_*HLenv*_*(k*,*l)* and *T*_*HLtrans*_*(k*,*l)* respectively) and thus represent the number of half-lives of the virus during that stage

$$C_{env}\left(k,l\right)=C_{HLenv}\left(k,l\right)\;*\;T_{HLenv}\left(k,l\right)$$

$$C_{trans}\left(j,k,l,m\right)=C_{HLtrans}\left(j,k,l,m\right)\;*\;T_{HLtrans}\left(j,k,l,m\right).$$

Thus in the baseline model

$$a(j,k,l,m)=2^{C_{env}(k,l)}\left(2^{C_{trans}(j,k,l,m)}C_{\min}-C_{env}(k,l)\right),$$ (8)

where *C*_*min*_ is a threshold viral load upon arrival at the EU MS, below which the product is considered not to be contaminated.

The default estimate for the prevalence of contamination in the raw products, *p*_*Graw*_*(k)*, is based on the estimated prevalence of active virus shedding in bats, *p*_*Binf*_*(k)*, the bat contact rate with the product, *p*_*Bcontact*_*(k)* and seasonality of the virus, i.e. the proportion of the year that bats can shed the virus, *p*_*season*_*(k)*

$$p_{Graw}\left(k\right)=p_{Binf}\left(k\right)*p_{Bcontact}\left(k\right)*p_{season}\left(k\right).$$ (9)

#### Live animals

There are a number of reasons why live animals may be brought into the EU, including livestock for production purposes, scientific research, for zoos and travelling personal companion animals. Animal species from an exporting country may become infected if they are susceptible and are exposed to an infectious dose of virus. Similar to humans, there may be a sub-clinical phase where these animals could arrive into the EU with undetected infection, or like bats in the case of NiV, they may be asymptomatic carriers.

Let *N*_*A*_*(s*,*j*,*k)* be the number of animals of species *s* from exporting country *k* to MS *j* in one year, then the probability at least one infected animal enters MS *j*, *P*_*A*_*(j)*, is given by

$$P_{A}(j)=1-\prod_{k=1}^{K}\prod_{s=1}^{S}\;{\left(1-p_{Ain}(s,k)\right)}^{N_{A}(s,j,k)},$$ (10)

where *S* is the total number of animal species considered and *p*_*Ain*_*(s*,*k)* the probability that an animal of species *s* in exporting country *k* will be infected. If the species is an asymptomatic carrier then the prevalence in the species in country *k*, is used, but if the species is thought to develop clinical symptoms then a similar method to *p*_*Hin*_*(s*,*k)* is used. Note we assume that animals showing clinical signs will not be imported. Let *p*_*Ainf*_*(s*,*k)* be the prevalence of virus in species *s* in country *k* and *T*_*AID*_*(s*,*k)* be the time to clinical signs in species *s*, then

$$\begin{array}{l} p_{Ain}(s,k)={\bar{\nu }}_{A}(s,k),\\ \nu_{A}(s,k)∼Gamma\left(\theta_{A}(s,k)\;*\;\frac{T_{AID}(s,k)}{365},1\right), \end{array}$$ (11)

where

$$\theta_{A}(s,k)=p_{A\inf}(s,k)\;*\;Min\left(1,\frac{T_{ADK}(s,k)}{365}\right)$$ (12)

and *T*_*ADK*_*(s*,*k)* is the average duration of time an animal of species *s* spends in country *k* (365 days if a native animal, same as human tourists, *T*_*DK*_*(k)*, if a companion animal).

#### Illegal bushmeat import

Bushmeat is a generic term to describe meat from a variety of wild animal species. It is generally illegal to bring such products into the EU. Bats are considered bushmeat, but only make up a small proportion of illegal seizures [32–35]. However, other species reported as bushmeat, such as non-human primates and duikers, are known to be susceptible to a number of bat-borne viruses [36–38]. The model does not include any effects of processing of bushmeat. There is evidence that bats are often smoked, which could reduce the viability of viral particles within the meat, but there was insufficient evidence to determine by how much and whether this would be sufficient to negate the risk of infection upon consumption, as supported by the European Food Safety Authority (EFSA) report [19]. Bushmeat can arrive at EU MSs by freight or post, but in this model we only consider the arrival of bushmeat in the luggage of aircraft passengers.

The probability of at least one contaminated bushmeat consignment entering MS *j* in a year, *P*_*B*_*(j)*, is given by

$$P_{B}(j)=1-\prod_{k=1}^{K}\prod_{s=1}^{S}\prod_{i=1}^{I}\;{\left(1-p_{BMin}(s,i,j,k)\right)}^{N_{H}(j,i,k)*p_{BMSp}(s)},$$ (13)

where *p*_*BMSp*_*(s)* denotes the proportion of all bushmeat that is from species *s* and *p*_*BMIn*_*(s*,*j*,*k)* is the probability of an individual consignment of contaminated bushmeat successfully making it into MS *j*. This is estimated by combining the probability of a passenger of type *i* bringing in bushmeat from exporting country *k*, *p*_*BM*_*(i*,*j*,*k)*, and the probability that a consignment of bushmeat is contaminated, *p*_*BMContam*_*(s*,*k)*

$$p_{BMin}(s,i,j,k)=p_{BM}(i,j,k)\;*\;p_{BMContam}(s,k).$$ (14)

The actual number of bushmeat consignments entering the EU from country *k* is estimated based on the number of bushmeat consignments seized in the EU MS, *N*_*seized*_*(i*,*j*,*k)*, and an under-reporting factor based on the proportion of passengers luggage that are searched, *p*_*UF*_*(j)*

$$N_{bm}(i,j,k)=N_{seized}(i,j,k)/p_{UF}(j).$$ (15)

Therefore, we estimate the probability of a passenger of type *i* attempting to bring in bushmeat from exporting country *k*, *p*_*BM*_*(i*,*j*,*k)*

$$p_{BM}(i,j,k)=\frac{N_{bm}(i,j,k)}{N_{H}(i,j,k)}.$$ (16)

## 4 Case Study: Nipah Virus

### 4.1 Parameterisation for NiV

A list of parameter estimates is given in Table 1. Further information on parameterisation is provided in S1 Appendix).

**Table 1:** Parameterisation of the generic framework for entry of bat-borne zoonotic viruses into the European Union: NIV case study.

Parameter	Description	Values	Reference
*k*	Exporting Country	Bangladesh (BGD), India (IND), Cambodia (CAM), East Timor (TMR), Indonesia (IDN), Malaysia (MAL), Singapore (SIN), Thailand (THA)	Assumed by authors
*n*_*Hinf*_*(k)*	Number of human infections in exporting country *k*, in one year	BGD = 27, IND = 66, all other countries = 0	[10, 39, 40]
*N*_*pop*_*(k)*	Population of country *k*	Variable	[41]
*P*_*Hinf*_*(k)*	Prevalence of human infection in country k	*nHinf(k)/Npop(k)*	
*P*_*BInf*_*(k)*	Prevalence of *active* bat infection in exporting country *k*	All countries = 0.2%	Assumed by author based on [9, 42–47]
*P*_*Ainf*_*(s*,*k)*	Prevalence of animal infection in species *s* in exporting country *k*	Assumed same as human infection, due to lack of animal specific data. Human prevalence considered better proxy than bat prevalence as infection in bats is asymptomatic.	Assumed by authors
*N*_*H*_*(i*,*j*,*k)*	Total passengers arriving at MS *j* from exporting country *k*	Variable	Eurostat dataset avia_paexcc [48]
*T*_*IP*_*(k)*	Average time to clinical symptoms of the virus	9 days for all countries	[49]
			
*i*	Passenger Type	Foreign Business (FB), Foreign holiday (FH), Foreign visit friends and relatives (FVFR), Member State business (MSB), Member State holiday (MSH), Member State visit friends and relatives (MSVFR), Miscellaneous (MISC)^*^, international connectors (CON)^**^	[50]
*P*_*assSplit*_*(i*,*j)*	Split of passengers between types	FB = 7.69%, FH = 7.69%, FVFR = 8.97%, MSB = 7.69%, MSH = 39.74%, MSVFR = 11.54%, MISC = 5.13%, CON = 11.54%	[50]
*T*_*DK*_*(i*,*k)*	Duration of time (days) passenger type *i* spends in exporting country *k*	{FB, FH, FVFR, CON} = 365	[50]
		MSB = 18, MSH = 21, MSVFR = 37, MISC = 48	
*l*	Legal Trade products	All products in FAOstat under section 8 –Fruits and derived products (60 products in total).	[51]
*N*_*G*_*(j*,*k*,*l*,*m)*	Volume (tonnes) of trade product, *l*, imported to MS *j* from exporting country *k*	Variable	[51]
*p*_*Bcontact*_*(k)*	Probability of bat contact with raw product	0.02	Estimated based on [52, 53]
*P*_*season*_*(k)*	Proportion of the year bats may shed active virus	1/3	[6]
*c*_*0*_*(x)*	Initial viral load on product	*c*_*0*_*(x) ~ LogNormal(a*,*b)*,	Estimated from [54]
		mean = 2 log_10_ TCID_50_/mm,	
		variance = 2.25 log_10_ TCID_50_/mm	
*C*_*HLenv*_*(k*,*l)*	Half-life of NiV in environment, pre-harvesting (hours)	6.15	Estimated based on [55]
*T*_*HLenv*_*(k*,*l)*	Duration of time spend in the environment	24 hours	Assumed by author
*C*_*proc*_*(k)*	Reduction in viral load (Log_10_ TCID_50_) due to processing method	Raw product– 0, Prepared product– 1, processed product– 2, chemically processed product– 3, thermal/high pressure treated product– 3, thermal/high pressure treated concentrated product– 4, sterilised product—4.	See supplementary material S1 Appendix
*C*_*HLtrans*_*(j*,*k*,*l*,*m)*	Half-life of NiV during transport (hours)	308	Estimated based on [56]
*T*_*HLtrans*_*(j*,*k*,*l*,*m)*	Duration of journey between exporting country and EU MS	Great circle distance (miles) / average speed of transport (mph).	Assumed by author
		Average speed is 25mph if *m* = sea and 500mph if *m* = air	
		Great circle distance estimated using spatial data from the wrld_simpl map in the maptools package in R	
*C*_*min*_	Minimum Viral load to consider product contaminated in EU MS	1 Log_10_ TCID_50_	Assumed by author
*s*	Live Animals species	*Canis familiaris* (dog), *Felis catus* (cat), *Mustela putorius furio* (ferret) and *Tapirus spp*. (tapir), *Sus scrofa domesticus* (domestic pig) and all non-human primates	Based on evidence of prior susceptibility to NiV [57–60]
*N*_*A*_*(s*,*j*,*k)*	Number of live animals of species *s*, imported to MS *j* from exporting country *k*	Variable	[61]
*T*_*ADK*_*(s*,*k)*	Duration of time animal of species type *s* spends in exporting country *k*	Native animal = 365 days	Assumed by author
		Companion animal = *T*_*DK*_*(MSH*,*k)*	
*p*_*BM*_*(i*,*j*,*k)*	probability of a passenger attempting to bring in bushmeat to MS *j*, from exporting country *k*	0 for *i =* {MSB,MSH}, otherwise 1.10209*10^−4^	Based on data from UK border force
*N*_*seized*_*(i*,*j*,*k)*	Number of bushmeat items seized in MS *j* from exporting country *k*	Variable	Based on data from UK border force
*P*_*UF*_*(j)*	Probability luggage is searched (i.e. under reporting factor)	0.005	[33]
*p*_*BMSp*_*(s)*	Probability bushmeat is of species *s*	1.5% Bats, 98.5% other species	[35]
*p*_*BMcontam*_*(s*,*k)*	Probability bushmeat is contaminated	*p*_*Binf*_*(k)* if species is bat, otherwise assumed equal to *p*_*Hinf*_*(k)*	Assumed by author

*Miscellaneous includes travelling for study, to attend sporting events, for shopping, health, religious or for other purposes, together with visits for more than one purpose when none predominates (e.g. business and holiday). Overseas visitors staying overnight en route to other destinations are also included. [50]

**International connectors is an estimated figure, consisting of passengers that are not travelling on a domestic flight and who have fallen outside the scope of the survey (e.g. transferred planes at a UK airport without clearing customs)

### 4.2 Scenario analyses for NiV

To assess the performance of the model and the effect of uncertainty surrounding model estimates, we considered a number of scenarios. These analyses use the NiV model as a baseline to investigate the impact on the risk to the EU when key parameters are altered. Only the scenarios which had the most impact on the baseline model results are reported here:

1. Inclusion of an annual probability of an outbreak of NiV in humans in country *k*, *p*_*ob*_*(k)*, so NiV infections don’t necessarily occur every year
  $$\begin{array}{l} n_{H\inf}(k,t)=n_{H\inf}(k)\;*\;P_{ob}(k,t)\\ P_{ob}(k,t)∼Binomial\left(1,p_{ob}(k)\right) \end{array},$$
  where, *t* is time (in years) and *n*_*Hinf*_*(k*,*t)* is the number of human cases in country *k* at time *t*.
2. There is one human case of NiV in every exporting country (which included countries such as Thailand, a popular tourist destination), *n*_*Hinf*_*(k)* = 1, to allow for the potential for (unreported) spill-over to humans in countries where infected bats are present. NB, we are not suggesting there is strong evidence for infection in all these countries; it is a ‘worst case’ scenario to investigate the adverse impact on the model outputs.
3. Reducing the prevalence of NiV in bats, *P*_*BInf*_*(k)*, by 50% (3a), 75% (3b), 90% (3c) and 97.5% (3d) and 99% (3e). The estimate for the prevalence in bats was considered relatively high (0.2%) given current data and modelling assumptions such as heterogeneity in the prevalence within the bat-populations. These reductions corresponded to bat prevalences of 0.1% (3a), 0.05% (3b), 0.02% (3c) 0.005% (3d) and 0.002% (3e).
4. Incorporate variability in the number of live animals in a consignment as the baseline model uses an average value for all consignments; *N*_*A*_*(s*,*j*,*k*,*c)~Multinomial(p*_*Avar*_*(s))*, where *c* is a consignment of species *s* and *p*_*Avar*_*(s)* is a vector of probabilities of a consignment containing *N*_*A*_ animals, as derived from the TRACES data.
5. Allowing all passenger types, *i*, to potentially bring in bushmeat to the EU at the same rate, *p*_*BM*_*(k*,*i)* = 1.1x10^-4^ for all *i;* the baseline model assumes that EU residents travelling on business or holiday will not bring back bushmeat.
6. Reducing the probability of bushmeat being of bat origin, *p*_*BMSp*_*(bat) =* 0.15%. The baseline estimate of 1.5% is based on a study conducted in the United States and may be an overestimate.
7. Include China as an exporting country with 28 human cases per year. This scenario is simply to investigate the impact if NiV were to spread to another country with a lot of trade and human travel links to the EU; there is no indication that NiV is actually present in China.
8. Remove grapes as an at risk trade product. This is a scenario designed simply to investigate the impact of a change in at risk product types.

## 5 Results

**Fig 2:**
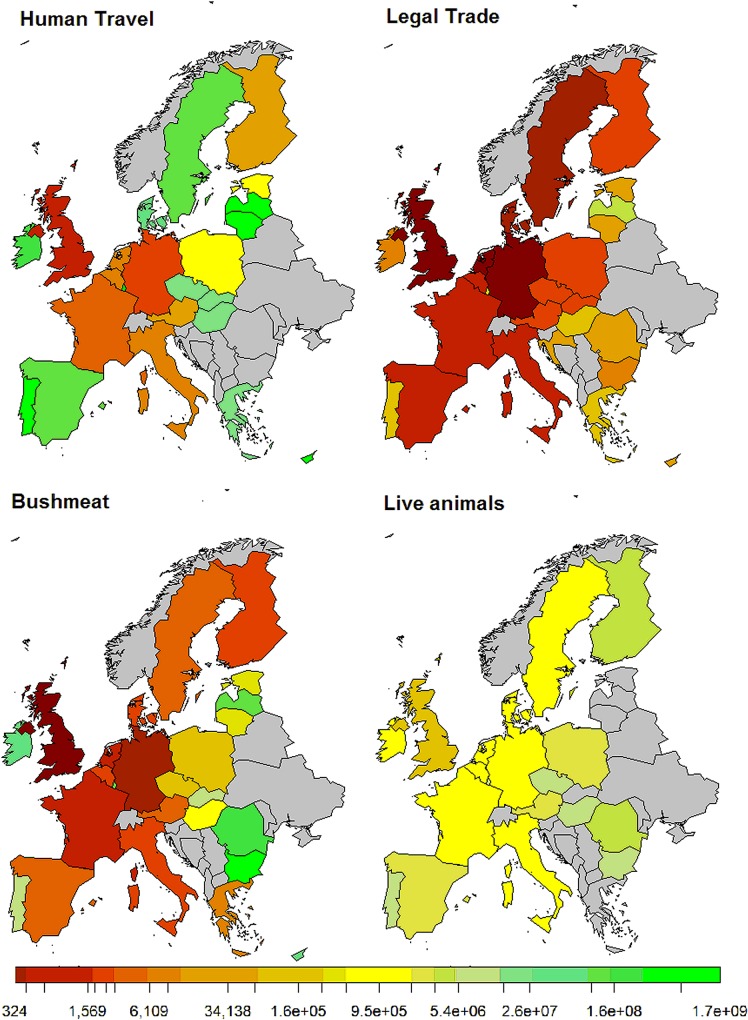
Average number of years until an introduction event of NiV, by EU MS and route. Colour scale from red to green, where red is the lower number of years before an introduction event.

At a MS level, the baseline model predicted the Netherlands and the United Kingdom to be the most likely points of introduction (Fig 2). The Netherlands was the most likely legal trade destination, while the UK was the most likely destination for introduction via human travel, bushmeat and live animals; although the risk from live animals was much lower than for other routes.

The baseline model results (Table 2) suggested that legal trade was the most likely route of introduction of NiV followed by bushmeat and human travel. The results from the scenario analyses show that the uncertainty and variability surrounding the model parameterisation, in particular the estimate for the prevalence in bats, can have considerable impact on the average number of years to an EU introduction of NiV, relative to the baseline model parameterisation (Table 2). However, the relative ranking between routes was more robust; the only scenarios to have an impact on the relative ranking between the routes were those involving much lower estimates for the bat prevalence.

**Table 2:** Average number of years to an introduction of NiV for different scenarios, via the individual routes and all routes combined. Note that further description of scenarios is provided in Section 4.2.

Scenario	Human travel	Legal trade	Bush-meat	Live animals	All routes
**Baseline**	540	12	70	51649	10
**1) Incorporate probability of outbreak**	2440	12	70	51649	10
**2) At least 1 human case in all exporting countries**	153	12	70	51649	10
**3a) Bat Infection 50% reduction**	540	23	139	51649	19
**3b) Bat Infection 75% reduction**	540	46	276	51649	37
**3c) Bat Infection 90% reduction**	540	115	682	51649	83
**3d) Bat Infection 97.5% reduction**	540	459	2596	51649	226
**3e) Bat Infection 99% reduction**	540	1147	5915	51649	344
**4) Vary number of live animals in consignment**	540	12	70	35062	10
**5) All passenger types carry bushmeat**	540	12	37	51649	9
**6) Prevalence of NiV in bat bushmeat = 0.15%**	540	12	682	51649	12
**7) Include China as an exporting country**	328	3	40	46039	3
**8) Remove Grapes as an at risk trade product**	540	37	70	51649	23

For scenarios 1–6, the relative rankings of the MSs were fairly robust. The top three MSs for all routes were always the UK, the Netherlands and Germany (Table 3). Between all 28 EU MSs, the largest variation in rank was 5 places, with Sweden falling from 5^th^ to 9^th^ in scenario 3e, when a much lower estimate of bat prevalence is used. As might be expected, there was greater variation in the ranking for scenarios 7–8, which involved changing the exporting countries and/or at risk trade products, with some MSs changing by as much as 15 places.

**Table 3:** Baseline relative ranking of MS risk of introduction of NiV from all routes, range (min–max ranking) over scenarios 1–6 and range over all scenarios (including removal of grapes and addition of China).

Baseline ranking	Member State	Scenarios 1–6: Range (min, max ranking)	All scenarios: Range	Baseline ranking	Member State	Scenarios 1–6: Range (min, max ranking)	All scenarios: Range
**1**	The Netherlands	1 (1, 2)	1	**15**	Ireland	1 (15, 16)	5
**2**	Great Britain	1 (1, 2)	1	**16**	Greece	5 (15, 20)	5
**3**	Germany	0 (3, 3)	1	**17**	Bulgaria	2 (16, 18)	5
**4**	France	4 (4, 8)	5	**18**	Estonia	2 (16, 18)	9
**5**	Sweden	5 (4, 9)	7	**19**	Cyprus	2 (18, 20)	9
**6**	Italy	1 (5, 6)	3	**20**	Romania	2 (29, 21)	15
**7**	Denmark	5 (5, 10)	7	**21**	Lithuania	1 (21, 22)	12
**8**	Belgium	2 (6, 8)	3	**22**	Croatia	3 (21, 24)	7
**9**	Finland	3 (7, 10)	7	**23**	Portugal	3 (20, 23)	4
**10**	Spain	2 (9, 11)	4	**24**	Malta	2 (24, 26)	5
**11**	Austria	3 (8, 11)	8	**25**	Hungary	2 (23, 25)	2
**12**	Slovakia	1 (12, 13)	10	**26**	Luxembourg	1 (25, 26)	1
**13**	Poland	1 (12, 13)	4	**27**	Latvia	0 (27, 27)	14
**14**	Czech Republic	0 (14, 14)	4	**28**	Slovenia	0 (28, 28)	1

## 6 Discussion

In this paper we have described the development of a risk assessment framework for the introduction of bat-borne viruses to the EU through a number of routes previously identified to be a risk, namely; human travel, legal trade (e.g. foodstuffs and products of animal origin), live animal movements and illegal importation of bushmeat. The model utilises large, freely available, global datasets on human travel, legal trade and live animal movements to assess the volume of traffic between individual countries of the world and EU MSs. The main strengths of this model approach lie in the comparison of the relative risk of introduction between EU MSs, running hypothetical future scenarios to investigate the impact of a change in factors such as trade patterns or human demographics, interrogating what data are available and suggesting areas where further research would be useful.

The model was parameterised for NiV, using what were considered the most likely parameter estimates. However, due to the scarcity of data on NiV for some of the model parameters there were inevitably data gaps in the model, in particular with regards to the prevalence of infection in animals and the probability of contamination of trade products in the exporting countries. As such, multiple scenario analyses were conducted, the results of which should be considered as plausible alternatives alongside the baseline results.

Results from the baseline NiV model suggested that legal trade was the most likely route of entry of NiV into the EU, followed by import of bushmeat, with human travel over 40-fold lower than these routes. The risk from pets and other live animals was very low in comparison to all other routes, likely because the model estimated that there were only around 200 relevant animals entering the EU from the exporting countries. The scenarios suggested that the relative ranking of the EU MSs and the routes of introduction were fairly robust to changes in the NiV parameter estimates, but were susceptible to changes in the exporting countries and at risk products considered. For example, in the scenario without grapes, Sweden dropped from 5^th^ to11^th^ and Estonia from 18^th^ to 25^th^, as it was their predominant fruit import from the exporting countries in question. This highlights the importance of the import data and how a change in demand for a product may alter the associated risk. An example of this was in May 2014 when the EU temporarily banned imports of mangoes from India after fruit flies were found in consignments [62].

The scenario analyses also highlighted that the absolute value estimates were highly susceptible to changes in the prevalence of NiV in bats in the exporting countries; due to the high uncertainty around this parameter estimate, further field studies are recommended to enable more robust estimates of NiV prevalence in bats. Another area of uncertainty is the future: the model estimates of time until an introduction event are based on the assumption that parameter values remain constant over time. However, this is unlikely to actually be the case; for example, there has been a continuing general increase in airline travel to EU countries over recent years, which if it were to continue could potentially impact both the human travel and bushmeat routes in the model. Therefore, the model should be updated with new data when available and results reassessed. Projections in future changes (growth or decline) in the parameters could be considered in future models to improve accuracy, although uncertainties around these may limit the added value of increasing the complexity of the model in this way; for example, while the general trend of an increase in airline travel may continue this may not necessarily be the case in the specific countries considered, due to factors such as social or political change.

Data for parameters such as volume of trade and human travellers were particularly sparse for Eastern European countries and it was not clear if this was representative or simply due to under-reporting. The level of detail known about the amount of bushmeat entering the EU is also highly uncertain, a finding echoed in a recent assessment by the EFSA [19]. We have assumed a high level of underreporting of illegal bushmeat, but if targeted testing is effective the actual level could be much lower. We assumed, based on a previous US study, that a fairly low proportion of bushmeat is from bats, 1.5% [35], but given the level of human travel from some countries in South East Asia this still results in an estimate of well over 1000 people bringing in bushmeat to some MSs each year. Coupled with our estimate of NiV prevalence in bats, which is higher than the estimated prevalence for other animals, this resulted in a higher risk for ‘bat’ compared to ‘non-bat’ bushmeat. It should be remembered that the model does not include effects of any processing of bushmeat, which could reduce the risk of contamination.

Validation of the model is difficult as there are few data on the risk of NiV introduction to the EU aside from the fact that NiV has not been detected in the EU to date. Our baseline results indicate that, if the modelled situation were to continue unchanged, it would likely be hundreds of years before NiV is introduced via human travel or live animals, which would be consistent with it never having been detected via these routes. The result that legal trade is the most likely route of introduction of NiV is in agreement with the results of a previous qualitative entry assessment of henipaviruses into the UK [20]. The model does not consider the exposure and consequence stages of a full risk assessment, which are also important in contextualising the results. For example, an introduction event via legal trade is unlikely to have the same impact (e.g. outbreak or establishment of disease) as an introduction event via human travel as such risks depend on the various exposure pathways upon importation of disease and the quantity to which the susceptible human/livestock is exposed via the different commodities. In this entry assessment model, any infected ‘connectors’ (i.e. passengers who will not leave the airport between connecting flights) constitute an introduction to the MS. If we were considering exposure and consequence, then the risk from these connectors may be much smaller than passengers who will leave the airport and enter the MS. However, there are data to suggest that a reasonable proportion of UK connectors are going onto other EU countries [44], highlighting the significant interconnectedness of the EU and that an introduction event into one MS poses a risk for the whole EU.

By design, the data used to parameterise the model are, in general, derived from information that are freely available for all countries. As such, the model does not consider some of the more complex issues with regards to virus transmission such as seasonality of fruit (the harvesting season of some products may not overlap with when bats are likely to shed virus), climacteric fruits (some fruits are deliberately picked unripe for export and, as such, are unlikely to be in contact with bats who generally prefer fully ripe fruit), the degree of overlap of fruit orchards with susceptible bat populations (affecting probability of bat contact with raw produce), individuals who may be engaging in high risk practices (e.g. healthcare workers, tourists visiting bat caves [63] and attending traditional funerals [1]) and homogeneity of virus distribution in the host country (NiV has predominantly been reported in areas close to rivers such as the Ganges, [27], and the seroprevalence of Ebola in Gabon is reportedly higher in forest areas as opposed to savannah [64]). Such in depth analysis could be suited to a country specific case-study and may be advisable to gain a more comprehensive understanding of some of the more specific variation in risk.

The model framework described here is a useful tool for initial, rapid, quantitative prediction of potential risk of introduction to the EU of zoonotic bat-borne viruses and, importantly, can provide an indication of which routes are of most importance to each individual MS. The model design is modular, so is relatively easy to update in light of new data, such as changes in trade patterns, human demographics or spillover of NiV to new competent bat species. Additional routes of entry, such as bat migration, that might be important for new diseases or become more important in the future can also be incorporated. The model is also designed to be adaptable to assess the risk of entry of other infectious organisms given the incorporated underlying global movement and trade of products and people.

## Supporting Information

S1 Appendix In-depth NiV parameterisation. (DOC)

## References

[1] WHO. Nigeria is now free of Ebola virus transmission 2014 [cited 2014 Dec 2014]. Available from: http://www.who.int/mediacentre/news/ebola/20-october-2014/en/index1.html.

[2] WHO. Ebola virus disease outbreak—West Africa 2014 [September 2014]. Available from: http://www.who.int/csr/don/2014_09_04_ebola/en/.

[3] Mari Saez A, Weiss S, Nowak K, Lapeyre V, Zimmermann F, Dux A, et al Investigating the zoonotic origin of the West African Ebola epidemic. EMBO molecular medicine. 2015;7(1):17–23. 10.15252/emmm.201404792 . 25550396 PMC4309665

[4] Swanepoel R, Smit SB, Rollin PE, Formenty P, Leman PA, Kemp A, et al Studies of reservoir hosts for Marburg virus. Emerging Infectious Diseases. 2007;13(12):1847–51. 10.3201/eid1312.071115 18258034 PMC2876776

[5] Leroy EM, Kumulungui B, Pourrut X, Rouquet P, Hassanin A, Yaba P, et al Fruit bats as reservoirs of Ebola virus. nature. 2005;438(7068):575–6. 10.1038/438575a . 16319873

[6] Wacharapluesadee S, Boongird K, Wanghongsa S, Ratanasetyuth N, Supavonwong P, Saengsen D, et al A longitudinal study of the prevalence of Nipah virus in *Pteropus lyle*i bats in Thailand: evidence for seasonal preference in disease transmission. Vector-Borne and Zoonotic Diseases. 2010;10(2):183–90. 10.1089/vbz.2008.0105 . 19402762

[7] Amman BR, Carroll SA, Reed ZD, Sealy TK, Balinandi S, Swanepoel R, et al Seasonal Pulses of Marburg Virus Circulation in Juvenile *Rousettus aegyptiacus* Bats Coincide with Periods of Increased Risk of Human Infection. Plos Pathogens. 2012;8(10). 10.1371/journal.ppat.1002877 . PMC3464226 23055920

[8] Memish ZA, Mishra N, Olival KJ, Fagbo SF, Kapoor V, Epstein JH, et al Middle East respiratory syndrome coronavirus in bats, Saudi Arabia. Emerging Infectious Diseases [Internet] 2013;19(11). 10.3201/eid1911.131172. PMC3837665 24206838

[9] Chua KB, Koh CL, Hooi PS, Wee KF, Khong JH, Chua BH, et al Isolation of Nipah virus from Malaysian Island flying-foxes. Microbes and Infection. 2002;4(2):145–51. 10.1016/s1286-4579(01)01522-2 . 11880045

[10] IEDCR. Nipah situation in 2014. Update on February 28, 2014 2014 [October 2014]. Available from: http://www.iedcr.org/pdf/files/nipah/Nipah%20Infection_27.02.2014.pdf.

[11] Luby SP, Gurley ES, Hossain MJ. Transmission of Human Infection with Nipah Virus. Clinical Infectious Diseases. 2009;49(11):1743–8. 10.1086/647951 . 19886791 PMC2784122

[12] Luby SP, Hossain MJ, Gurley ES, Ahmed BN, Banu S, Khan SU, et al Recurrent Zoonotic Transmission of Nipah Virus into Humans, Bangladesh, 2001–2007. Emerging Infectious Diseases. 2009;15(8):1229–35. 10.3201/eid1508.081237 . 19751584 PMC2815955

[13] Cutler SJ, Fooks AR, van der Poel WHM. Public Health Threat of New, Reemerging, and Neglected Zoonoses in the Industrialized World. Emerging Infectious Diseases. 2010;16(1):1–7. 10.3201/eid1601.081467 . 20031035 PMC2874344

[14] ECDC. Updated rapid risk assessment. Severe respiratory disease associated with Middle East respiratory syndrome coronavirus (MERS-CoV). Ninth update, 24 April 2014 2014 [October 2014]. Available from: http://www.ecdc.europa.eu/en/publications/Publications/Middle-East-respiratory-syndrome-coronavirus-risk-assessment-25-April-2014.pdf.

[15] ECDC. Rapid risk assessment. Outbreak of Ebola virus desease in West Africa. Third update, 1 August 2014 2014 [October 2014]. Available from: http://www.ecdc.europa.eu/en/publications/Publications/ebola-outbreak-west-africa-1-august-2014.pdf.

[16] PHE. Risk assessment of the Ebola outbreak in West Africa: Updated 19th Septemeber 2014 2014a [October 2014]. Available from: https://www.gov.uk/government/uploads/system/uploads/attachment_data/file/356663/Ebola_Risk_Assessment_update_19_Sept.pdf.

[17] WHO. WHO risk assessment. Human infections with Zaïre Ebolavirus in West Africa. 24 June 2014 2014 [October 2014]. Available from: http://www.who.int/csr/disease/ebola/EVD_WestAfrica_WHO_RiskAssessment_20140624.pdf.

[18] Gomes MFC, Pastore y Piontti A, Rossi L, Chao D, Longini I, Halloran ME, et al Assessing the International Spreading Risk Associated with the 2014 West African Ebola Outbreak. PLOS Currents Outbreaks. 2014 10.1371/currents.outbreaks.cd818f63d40e24aef769dda7df9e0da5 25642360 PMC4169359

[19] EFSA. An update on the risk of transmission of Ebola virus (EBOV) via the food chain. EFSA Journal. 2014;12(11):3884.

[20] Snary EL, Ramnial V, Breed AC, Stephenson B, Field HE, Fooks AR. Qualitative Release Assessment to Estimate the Likelihood of Henipavirus Entering the United Kingdom. Plos One. 2012;7(2). 10.1371/journal.pone.0027918 . PMC3273481 22328916

[21] OIE. Handbook on Import Risk Analysis for Animals and Animal Products Paris, France:OIE 2004.

[22] Goddard AD, Donaldson NM, Horton DL, Kosmider R, Kelly LA, Sayers AR, et al A Quantitative Release Assessment for the Noncommercial Movement of Companion Animals: Risk of Rabies Reintroduction to the United Kingdom. Risk Analysis. 2012;32(10):1769–83. 10.1111/j.1539-6924.2012.01804.x . 22486335

[23] Jones RD, Kelly L, Fooks AR, Wooldridge M. Quantitative risk assessment of rabies entering Great Britain from North America via cats and dogs. Risk Analysis. 2005;25(3):533–42. 10.1111/j.1539-6924.2005.00613.x . 16022688

[24] de Vos C, Hoek, M., Fischer, E., de Koeijer, A., Bremmer, J. Risk Assessment Framework for Emerging Vector-Borne Livestock Diseases: Central Veterinary Institute, Wageningen, Report: 11-CVI0168 [April 2015]. Available from: http://library.wur.nl/WebQuery/clc/1982157.

[25] Defra. Exotic Animal Disease Risk Pathways & Countermeasures 2009 [April 2015]. Available from: https://www.gov.uk/government/uploads/system/uploads/attachment_data/file/69427/pb13567-risk-pathways-countermeasures-100310.pdf.

[26] Kelly L, Brouwer A, Wilson A, Gale P, Snary E, Ross D, et al Epidemic Threats to the European Union: Expert Views on Six Virus Groups. Transbound Emerg Dis. 2013;60(4):360–9. 10.1111/j.1865-1682.2012.01355.x . 22762483

[27] Simons RRL, Gale P, Horigan V, Snary EL, Breed AC. Potential for Introduction of Bat-Borne Zoonotic Viruses into the EU: A Review. Viruses-Basel. 2014;6(5):2084–121. 10.3390/v6052084 . PMC4036546 24841385

[28] Tatem AJ, Rogers DJ, Hay SI. Global transport networks and infectious disease spread In: Hay SI, Graham A, Rogers DJ, editors. Advances in Parasitology, Vol 62: Global Mapping of Infectious Diseases: Methods, Examples and Emerging Applications. Advances in Parasitology. 62 San Diego: Elsevier Academic Press Inc; 2006 p. 293–343. 10.1016/S0065-308X(05)62009-X PMC3145127 16647974

[29] Rahman MA, Hossain MJ, Sultana S, Homaira N, Khan SU, Rahman M, et al Date Palm Sap Linked to Nipah Virus Outbreak in Bangladesh, 2008. Vector-Borne and Zoonotic Diseases. 2012;12(1):65–72. 10.1089/vbz.2011.0656 . 21923274

[30] Luby SP, Rahman M, Hossain MJ, Blum LS, Husain MM, Gurley E, et al Foodborne transmission of Nipah virus, Bangladesh. Emerging Infectious Diseases. 2006;12(12):1888–94. 10.3201/eid1212.060732 17326940 PMC3291367

[31] EUROPHYT. European Union Notification System for Plant Health Interceptions—EUROPHYT: European Commission; 2014 [October 2014]. Available from: http://ec.europa.eu/food/plant/plant_health_biosafety/europhyt/index_en.htm.

[32] Chaber A-L, Allebone-Webb S, Lignereux Y, Cunningham AA, Rowcliffe JM. The scale of illegal meat importation from Africa to Europe via Paris. Conservation Letters. 2010;3(5):317–23. 10.1111/j.1755-263X.2010.00121.x .

[33] Falk H, Duerr S, Hauser H, Wood K, Tenger B, Loertscher M, et al Illegal import of bushmeat and other meat products into Switzerland on commercial passenger flights. Revue Scientifique Et Technique-Office International Des Epizooties. 2013;32(3):727–39. . 10.20506/rst.32.2.2221 24761726

[34] Telegraph. T. Frozen porcupines and bats confiscated in Paris exotic food raid 2013 [cited 2014 January 2014]. Available from: http://www.telegraph.co.uk/news/worldnews/europe/france/10500982/Frozen-porcupines-and-bats-confiscated-in-Paris-exotic-food-raid.html.

[35] Bair-Brake H, Bell T, Higgins A, Bailey N, Duda M, Shapiro S, et al Is That a Rodent in Your Luggage? A Mixed Method Approach to Describe Bushmeat Importation into the United States. Zoonoses Public Health. 2014;61(2):97–104. 10.1111/zph.12050 . 23678947

[36] Weingartl HM, Embury-Hyatt C, Nfon C, Leung A, Smith G, Kobinger G. Transmission of Ebola virus from pigs to non-human primates. Nature. 2012;2(811):1 10.1038/srep00811 23155478 PMC3498927

[37] Geisbert TW, Mire CE, Geisbert JB, Chan Y-P, Agans KN, Feldmann F, et al Therapeutic Treatment of Nipah Virus Infection in Nonhuman Primates with a Neutralizing Human Monoclonal Antibody. Science Translational Medicine. 2014;6(242). 10.1126/scitranslmed.3008929 . PMC4467163 24964990

[38] Rouquet P, Froment JM, Bermejo M, Kilbourn A, Karesh W, Reed P, et al Wild animal mortality monitoring and human Ebola outbreaks, Gabon and Republic of Congo, 2001–2003. Emerging Infectious Diseases. 2005;11(2):283–90. 10.3201/eid1102.040533 15752448 PMC3320460

[39] Chadha MS, Comer JA, Lowe L, Rota PA, Rollin PE, Bellini WJ, et al Nipah virus-assodiated encephalitis outbreak, Siliguri, India. Emerging Infectious Diseases. 2006;12(2):235–40. 10.3201/eid1202.051247 16494748 PMC3373078

[40] Arankalle VA, Bandyopadhyay BT, Ramdasi AY, Jadi R, Patil DR, Rahman M, et al Genomic Characterization of Nipah Virus, West Bengal, India. Emerging Infectious Diseases. 2011;17(5):907–9. 10.3201/eid1705.100968 . 21529409 PMC3321761

[41] CIA. Country populations July 2013 2013 [cited 2013 July 2013]. Available from: https://www.cia.gov/library/publications/the-world-factbook/rankorder/rawdata_2119.txt.

[42] Rahman SA, Hassan SS, Olival KJ, Mohamed M, Chang LY, Hassan L, et al Characterization of Nipah virus from naturally infected *Pteropus vampyrus* bats, Malaysia. Emerging Infectious Diseases. 2010;16(12):1990–3. 10.3201/eid1612.091790 . 21122240 PMC3294568

[43] Reynes JM, Counor D, Ong S, Faure C, Seng V, Molia S, et al Nipah virus in lyle's flying foxes, Cambodia. Emerging Infectious Diseases. 2005;11(7):1042–7. 10.3201/eid1107.041350 16022778 PMC3371782

[44] Johara MY, Field H, Rashdi AM, Morrissy C, van der Heide B, Rota P, et al Nipah virus infection in bats (order Chiroptera) in peninsular Malaysia. Emerging Infectious Diseases. 2001;7(3):439–41. 10.3201/eid0703.010312 11384522 PMC2631791

[45] Yadav PD, Raut CG, Shete AM, Mishra AC, Towner JS, Nichol ST, et al Short report: detection of Nipah virus RNA in fruit bat (*Pteropus giganteus*) from India. American Journal of Tropical Medicine and Hygiene. 2012;87(3):576–8. 10.4269/ajtmh.2012.11-0416 . 22802440 PMC3435367

[46] Shirai J, Sohayati AL, Mohamed Ali AL, Suriani MN, Taniguchi T, Sharifah SH. Nipah virus survey of flying foxes in Malaysia. Jarq-Japan Agricultural Research Quarterly. 2007;41(1):69–78. .

[47] Breed AC, Meers J, Sendow I, Bossart KN, Barr JA, Smith I, et al The Distribution of Henipaviruses in Southeast Asia and Australasia: Is Wallace's Line a Barrier to Nipah Virus? Plos One. 2013;8(4). 10.1371/journal.pone.0061316 . PMC3634832 23637812

[48] Eurostat. statistics database 2014 [January 2014]. Available from: http://epp.eurostat.ec.europa.eu/portal/page/portal/statistics/search_database.

[49] Hossain MJ, Gurley ES, Montgomery JM, Bell M, Carroll DS, Hsu VP, et al Clinical presentation of Nipah virus infection in Bangladesh. Clinical Infectious Diseases. 2008;46(7):977–84. 10.1086/529147 . 18444812

[50] CAC. Recent trends in growth of UK air passenger demand. 2014 [September 2014]. Available from: http://www.caa.co.uk/docs/589/erg_recent_trends_final_v2.pdf.

[51] FaoStat. trade data 2014 [Januray 2014]. Available from: http://faostat.fao.org/site/342/default.aspx.

[52] Khan SU, Gurley ES, Hossain MJ, Nahar N, Sharker MAY, Luby SP. A Randomized Controlled Trial of Interventions to Impede Date Palm Sap Contamination by Bats to Prevent Nipah Virus Transmission in Bangladesh. Plos One. 2012;7(8):7 10.1371/journal.pone.0042689 . PMC3414453 22905160

[53] Nahar N, Mondal UK, Hossain MJ, Uddin Khan MS, Sultana R, Gurley ES, et al Piloting the promotion of bamboo skirt barriers to prevent Nipah virus transmission through date palm sap in Bangladesh. Global health promotion. 2014;epub ahead of print. 10.1177/1757975914528249 PMC4666517 24755262

[54] de Wit E, Prescott J, Falzarano D, Bushmaker T, Scott D, Feldmann H, et al Foodborne Transmission of Nipah Virus in Syrian Hamsters. Plos Pathogens. 2014;10(3). 10.1371/journal.ppat.1004001 . PMC3953481 24626480

[55] Fogarty R, Halpin K, Hyatt AD, Daszak P, Mungall BA. Henipavirus susceptibility to environmental variables. Virus Research. 2008;132(1–2):140–4. 10.1016/j.virusres.2007.11.010 . 18166242 PMC3610175

[56] Scanlan JC, Kung N.Y., Selleck P.W., Field H.E. Survival of Hendra Virus in the Environment: Modelling the Effect of Temperature. Eco Health. 2014;Published online. 10.1007/s10393-014-0920-4 24643861 PMC7087565

[57] Clayton BA, Middleton D, Bergfeld J, Haining J, Arkinstall R, Wang L, et al Transmission routes for nipah virus from Malaysia and Bangladesh. Emerging Infectious Diseases. 2012;18(12):1983–93. 10.3201/eid1812.120875 . 23171621 PMC3557903

[58] Middleton DJ, Westbury HA, Morrissy CJ, van der Heide BM, Russell GM, Braun MA, et al Experimental Nipah virus infection in pigs and cats. Journal of Comparative Pathology. 2002;126(2–3):124–36. 10.1053/jcpa.2001.0532 . 11945001

[59] Mills JN, Alim ANM, Bunning ML, Lee OB, Wagoner KD, Amman BR, et al Nipah Virus Infection in Dogs, Malaysia, 1999. Emerging Infectious Diseases. 2009;15(6):950–2. 10.3201/eid1506.080453 . 19523300 PMC2727347

[60] Geisbert TW, Daddario-DiCaprio KM, Hickey AC, Smith MA, Chan Y-P, Wang L-F, et al Development of an Acute and Highly Pathogenic Nonhuman Primate Model of Nipah Virus Infection. Plos One. 2010;5(5). 10.1371/journal.pone.0010690 . PMC2872660 20502528

[61] TRACES. Trade Control and Expert System 2014 [January 2014]. Available from: https://webgate.ec.europa.eu/sanco/traces/.

[62] Defra. DEFRA welcomes end to restrictions on Indian mangoes 2015 [cited 2015 June 2015]. Available from: https://www.gov.uk/government/world-location-news/defra-welcomes-end-to-restrictions-on-indian-mangoes.

[63] WHO. Case of Marburg Haemorrhagic Fever imported into the Netherlands from Uganda 2008 [January 2014]. Available from: http://www.who.int/csr/don/2008_07_10/en/.

[64] Nkoghe D, Padilla C, Becquart P, Wauquier N, Moussavou G, Akue JP, et al Risk Factors for Zaire ebolavirus-Specific IgG in Rural Gabonese Populations. Journal of Infectious Diseases. 2011;204:S768–S75. 10.1093/infdis/jir344 . 21987749

