# Supplementary material for: A Generic Quantitative Risk Assessment Framework for the Entry of Bat-Borne Zoonotic Viruses into the European Union
Source: PLoS One. 2016 Oct 27;11(10):e0165383. doi: 10.1371/journal.pone.0165383 (PMC5082878; doi:10.1371/journal.pone.0165383)
Supplement: S1 Appendix — (DOC) [file pone.0165383.s001.doc]

**Appendix S1: In depth NiV parameterisation.**

**Overview**

Here we provide more detail on the parameterisation of the model.

**General Parameters**

*Exporting Countries, k.* The exporting countries were determined based on peer reviewed publications of where NiV had been identified in humans or animals. For bats, only positive results for live virus or RNA positive were considered. The list of exporting countries and references are listed in Table 1. Human cases in Malaysia and Singapore were excluded as the last case occurred >10 years ago; it was assumed that these countries were no longer likely to have NiV outbreaks on that scale.

Table 1: List of exporting countries for the NiV case study and reason for inclusion

| **Exporting Country (k)** | **Reason for inclusion** | **Reference** |
| --- | --- | --- |
| Bangladesh | Recent human cases of NiV | [1] |
| India | Recent human cases of NiV | [2,3] |
| Malaysia | NiV Identified in bats, previous human cases | [4-6] |
| Singapore | previous human cases | [7] |
| Cambodia | NiV Identified in bats | [8] |
| East Timor | NiV Identified in bats | [9] |
| Indonesia | NiV Identified in bats | [10] |
| Thailand | NiV Identified in bats | [11] |

**Human travel parameters**

*Number of human infections in exporting country, n_Hinf_(k).* NiV-Bangladesh in humans has, so far, been restricted to South-East Asia particularly Bangladesh and India. Since the first identified outbreak in 2001, disease is reported annually in multiple districts of Bangladesh. In 2014, there were 27 reported cases in Bangladesh [1], which we use as our estimate. India reported sporadic outbreaks in 2001 and 2007, both situated near the border with Bangladesh [2,3]. These two outbreaks resulted in a total of 71 cases and 50 deaths. The seasonal outbreaks which occur can be sporadic and infect many people. Thus we chose to model a ‘worst case scenario’ where an outbreak has occurred in India in the year and assume the number of human cases to be 66, the number of cases in the worst outbreak in India. We assume there are no human cases in any country other than Bangladesh and India. However, to account for the uncertainty about this due to underreporting we run scenarios allowing for 1 case per year in all other exporting countries (i.e. those with reported NiV antibodies or virus isolation in bats, but not humans).

*Probability of an outbreak, p_ob_(k,t),*The baseline model assumes a ‘worst case’ scenario where there is an outbreak in every country with reported human cases every year. The scenario analysis incorporates the probability of an outbreak per year, *p_ob_(k)*. For Bangladesh there are reported outbreaks every year, so *p_ob_(k)=*1. For India there have only been two identified outbreaks since the year 2000, thus *p_ob_(k)=*1/7. For all other countries, *p_ob_(k)=*0, as there have been no human cases reported since 2000.

*Number of passengers, N_H_(j,k).* Data on the total passengers on board an aircraft and the total passengers carried*[[1]](#footnote-2)* arriving at MS *j* from exporting country *k* are available from the Eurostat database *avia_paexcc* [12]. We use the passengers on board data as it does not include passengers boarding the plane at the EU MS. It does include direct transit passengers, but they will be on board when the plane stops in the MS and thus will have entered the MS. It is possible this figure might include passengers who board the plane between the exporting country and the EU MS, but we have no way of knowing this and excluding them from the dataset. Data from Eurostat on other forms of travel (maritime, road and rail) are also available, but there are little to no passengers recorded using these routes for the majority of countries not sharing a land or sea border with European countries, as is the case with all the exporting countries for NiV.

*Passenger Type, i. Table 2* shows data from a UK report that gives a breakdown of air travel from 2006, by the reason for the flight [13]. Due to lack of information regarding other EU MSs, these data are used to split the total passenger numbers from exporting country *k,* as obtained from the Eurostat database into reason for travel. In doing so, it is assumed that the percentage splits are the same for the UK as other EU MSs. This may overestimate international connectors for MSs without major airports that act as hubs, such as Heathrow. However, it was determined that while a more accurate breakdown would be beneficial for a detailed case-study risk assessment, it was unnecessary for this generic model.

**Table 2: Data on breakdown of total passengers by passenger type, taken from Civil aviation authority report figure on page 19 [13]**.

| **Passenger Type (*i*)** | **% Passengers of type *i*** | **% Passengers of type *i***  **(not including Domestic)** |
| --- | --- | --- |
| Domestic | 22 | NA |
| international foreign business (IFB) | 6 | 7.69 |
| international foreign holiday (IFH) | 6 | 7.69 |
| international foreign VFR^*^ (IVFR) | 7 | 8.97 |
| international UK business (MSB) | 6 | 7.69 |
| international UK holiday (MSH) | 31 | 39.74 |
| international UK VFR^*^  (MSVFR) | 9 | 11.54 |
| IPS miscellaneous^**^ (misc) | 4 | 5.13 |
| international connectors^+^ (con) | 9 | 11.54 |

*VFR=visiting friends or relatives

**IPS Miscellaneous includes travelling for study, to attend sporting events, for shopping, health, religious or for other purposes, together with visits for more than one purpose when none predominates (e.g. business and holiday). Overseas visitors staying overnight in the UK en route to other destinations are also included [13]

+International connectors is an estimated figure, consisting of passengers that are not travelling on a domestic flight and who have fallen outside the scope of the IPS (e.g. transferred planes at a UK airport without clearing customs).

*Duration of stay in exporting country, T_DK_(i,k). Table 3* shows UK data on duration of stay in foreign countries, broken down by passenger type [13]. These data do not exist for every country and so are broken down by region rather than country. There are issues with applying UK duration of stay to other EU MSs, not least of which is that some countries have particular ties (e.g. commonwealth countries with the UK, South American countries with Spain or Portugal and some African countries with France and the Netherlands). These ties may affect duration of stay. Similar data are available from Eurostat, but many MSs suffer from significant data gaps so it was decided not to use them.

**Table 3: Average duration of stay (days) of UK tourists in foreign countries, broken down by passenger type, derived from [13]**.

| **Region** | **Duration of stay (days) by passenger type** | | | | |
| --- | --- | --- | --- | --- | --- |
|  | **Holiday visits (MSH)** | **Business visits (MSB)** | **Visiting friends or relatives (MSVFR)** | **Miscellaneous (misc)** | **All visits** |
| North America | 15 | 9 | 18 | 25 | 15 |
| Europe | 8 | 4 | 10 | 8 | 8 |
| Other North Africa | 24 | 14 | 33 | 183 | 32 |
| South Africa | 22 | 12 | 22 | 28 | 21 |
| Other Africa | 16 | 20 | 29 | 38 | 22 |
| Other Middle East | 21 | 20 | 33 | 17 | 26 |
| Other Asia | 21 | 18 | 37 | 48 | 28 |
| Australia | 43 | 32 | 35 | 111 | 40 |
| Other Caribbean | 15 | 19 | 31 | 14 | 17 |
| Other Central & South. America | 29 | 11 | 23 | 34 | 25 |
| Rest of the World | 13 | . | 14 | 1 | 13 |
| Total World | 10 | 6 | 15 | 14 | 10 |

*Time to clinical signs, T_IP_(k).* Data from secondary cases of NiV in Bangladesh place the median incubation period at 9 days with a range of 6-11 days [14]. However, the incubation period following a single intake of raw date palm sap is reported to be 7 days, with a range of 2-12 days [1]. For this model, while the raw date palm sap is the more likely route of infection, we use the median value of 9 days as a worst case scenario.

*Population of country k, N_pop_(k).* Data on the population of countries were obtained from the CIA website [15]. Note that the estimates used were for June 2013, data from this website will be updated over time.

**Legal Trade import**

*Legal Trade Products*, *l.* Fruit bats are considered the reservoir for NiV and are known to consume various fruit crops produced for human consumption [16-18]. There is the risk that infected bats may contaminate raw fruit via saliva or urine whilst foraging for food; NiV-Malaysia has been isolated from raw fruit in the environment [5]. Thus, for NiV, all products in the FAOStat database recorded under section 8 – Fruits and derived products, were considered. As pigs are susceptible to NiV-Malaysia, products of pig origin were also considered, but as discussed in a previous paper there are little to no imports of these products to the EU [19], so they are not considered in this risk assessment.

Table 4 details the legal trade products considered in the risk assessment and the associated code in FAOStat.

Table 4: List of trade products group considered in the NiV case study and the associated code in FAOstat.

| **Trade Product** | **Item code** | **FCL Title** | **FCL Item code** |
| --- | --- | --- | --- |
| Bananas | 0486 | Juice of plum, concentrated | 0539 |
| Plantains | 0489 | Stone fruit, fresh nes | 0541 |
| Oranges | 0490 | Pome fruit nes | 0542 |
| Juice of Orange | 0491 | Strawberries | 0544 |
| Orange Juice, Concentrated | 0492 | Raspberries | 0547 |
| Tangerines, mandarins, clementines, satsumas | 0495 | Gooseberries | 0549 |
| Juice of Tangerine, Mandarin and Clementin | 0496 | Currants | 0550 |
| Lemons and limes | 0497 | Blueberries | 0552 |
| Juice of Lemon | 0498 | Cranberries | 0554 |
| Lemon Juice, Concentrated | 0499 | Berries nes | 0558 |
| Grapefruit and pomelo | 0507 | Grapes | 0560 |
| Juice of Grapefruit | 0509 | Juice of Grape | 0562 |
| Grapefruit Juice, Concentrated | 0510 | Vermouths, etc. | 0565 |
| Citrus fruit nes | 0512 | Marc of Grape | 0566 |
| Juice of Citrus Fruit nes | 0513 | Watermelons | 0567 |
| Citrus Juice, Concentrated nes | 0514 | Melons, Cantaloupes | 0568 |
| Apples | 0515 | Figs | 0569 |
| Cider, etc. | 0517 | Figs, Dried | 0570 |
| Apple Juice | 0518 | Mangoes | 0571 |
| Apple Juice, Concentrated | 0519 | Avocados | 0572 |
| Pears | 0521 | Pineapples | 0574 |
| Quinces | 0523 | Juice of Pineapples | 0576 |
| Apricots | 0526 | Dates | 0577 |
| Apricots, Dried | 0527 | Juice of Pineapples, Concentrated | 0580 |
| Sour cherries | 0530 | Juice of Mango | 0583 |
| Cherries | 0531 | Mango Pulp | 0584 |
| Peaches and nectarines | 0534 | Persimmons | 0587 |
| Plums | 0536 | Cashewapple | 0591 |
| Plums, dried | 0537 | Kiwi fruit | 0592 |
| Juice of plum | 0538 | Papayas | 0600 |

*Bat infection prevalence in exporting country, p_Binf_(k).* The prevalence of bat infection is based on information from peer reviewed publications on isolation of active virus in South-East Asia, including results for NiV-Malaysia (see Table 5). It is only relevant to consider bats that are actively shedding the virus (e.g. through saliva or urine), as they are the only ones able to contaminate products and the environment, or cause infection in other animals. It can be seen from Table 5 that there are few studies that fit these criteria, and of those that do, there are considerable differences regarding sampling method, sample type and sensitivity of the test, among other variables. It is clear that these data are not sufficient to accurately describe the variability and uncertainty in NiV prevalence in bats throughout South-East Asia, let alone at a country level, and any detailed parameterisation would only be describing the variability and uncertainty in the data, not in the actual bat prevalence. Additionally, due to the issue of selection bias in published studies, i.e. studies are more likely to be conducted in areas where NiV is thought to be present and studies that do not find virus are less likely to be published, it is thought that any estimate based on these data will likely be an overestimate. Thus, based on these data and expert opinion, we assume that the prevalence of active virus shedding in bats is *p_Binf_(k)=*0.2%. As this estimate is highly uncertain it is investigated extensively in the sensitivity analysis.

**Table 5: Summary of studies where NiV isolation has been attempted, detailing country of testing, type of samples taken, number of samples taken and number of positive tests.**

| **Country** | **Sample taken** | **Number tested** | **Number positive** | **Prevalence** | **Reference** |
| --- | --- | --- | --- | --- | --- |
| Malaysia | urine or urogenital swab | 272 | 1 | 0.37% | [4] |
| Malaysia | pooled urine | 588 | 2 | 0.34% | [5] |
| Cambodia | pooled urine | 769 | 2 | 0.26% | [8] |
| Malaysia | kidney/spleen tissue | 296 | 0 | 0 | [20] |
| India | liver/kidney homogenate | 140 | 0 | 0 | [21] |
| Malaysia | tissue homogenate | 270 | 0 | 0 | [22] |
| Indonesia | Urine | 62 | 0 | 0 | [9] |

*Volume of goods, N_G_(j,k,l).* Data on the volume (tonnes) of trade product *l* imported to MS *j* from exporting country *k, N_G0_(j,k,l),* is available from the FaoStat database*,* [23]. Additional data, provides information on the proportion of goods transported by each mode of transport (i.e. air and sea), *p_transG_(m)*. Thus, *N_G_(j,k,l,m)=N*_G0_*(j,k,l)*p_transG_(m)*.

*Seasonal infection of bats with NiV, p_BinfYr_(k).* Human infection of NiV in Bangladesh is seasonal, with cases generally occurring between December – March [1]. This coincides with the birthing season of *P. giganteus,* which has been reported to be between December-January with parturition in late May [24]. As human cases are often linked to consumption of date palm sap, which is thought to be contaminated by bats, this suggests a seasonal pattern of NiV infection in bats. This hypothesis is backed up by a longitudinal study on *P. lylei* bats conducted in Thailand, which only isolated the Bangladesh strain of Nipah Virus from bats during April-May, coinciding with the birthing season of *P. lylei* bats in Thailand [25]. Note that the birthing season of *P. giganteus* is not considered to be the same as *P. lylei*. Thus, we set *p_BinfYr_(k)=*1/3.

*Contact rate of bats with raw product, p_Bcontact_(k)*. There are no accurate data to estimate the bat contact rate, which is very variable depending on whether there is an intervention measure in place and the efficacy of that measure. One study observed bats in Bangladesh and concluded that the bats contacted date palm trees on 85% of the study nights [26]. This contact rate goes down to 35% when an intervention using skirts was used. A follow-up study with a better intervention using larger skirts reduced this rate to 2% of nights. However, it is not clear how often these interventions are employed in general. Another study, estimated that 15% of date palm sap harvesters in Bangladesh consistently used skirts throughout the sap collection season [27]. For the purpose of this study we use these data as a proxy for bat contact with raw fruit. We assume producers of products destined for the EU operate to high phytosanitary conditions and have high uptake of high quality control measures. Thus, we set *p_Bcontact_(k)=*0.02.

It is possible that susceptible bat species are only present in part of a country and thus some orchards may not have contact with bats at all. However, there is evidence that the *P. giganteus* species is present throughout Bangladesh, albeit in greater numbers in some areas [28]. This supports our default hypothesis that all orchards are exposed to bat contact. We acknowledge that there is likely to be variation in contact rate between fruit species but due to data availability and desire for a parsimonious model, we do not consider differentiation between fruit species in this model.

*Initial concentration on raw product, c_0_(x).* Unfortunately, there are no data available on NiV viral loads in bat saliva or urine from natural infection. However, experimental studies on NiV infection in hamsters and ferrets suggest that the amounts of virus shed are quite low, even when experimentally infected with high doses [29,30]. In one study pharyngeal viral loads were estimated from hamsters fed artificial date palm sap containing 5*10^8^ TCID_50_ NiV-Bangladesh using TCID_50_ equivalents determined by RT-PCR [31]. Based on these data we assume that the initial viral load on the raw product follows a log normal distribution with mean 2 log_10_ TCID_50_ eq/ml and standard deviation 1.6 log_10_ TCID_50_ eq/ml (variance=2.25 log_10_ TCID_50_ eq/ml). As these data are from experimental studies, the ingested dose of NiV is likely greater than that received in the field and so the estimate for amount excreted is likely an overestimate.

*Duration of time spend in the environment, T_HLenv_(k,l).* There is evidence to suggest that there will be at least a short period of time between the initial contamination and further processing where raw produce will be stored at ambient temperatures ([32-34]). This is an important period as NiV will decay faster at higher temperatures, i.e. in the absence of a cold chain [35]. However, there is a lack of data on exactly how long this period could be; the authors assume a period of 24 hours.

*Virus decay in environment, C_HL_(u,j,k,l,m).* Previous experimental research estimated the half-life of NiV-Malaysia in the urine of *Pteropus vampyrus* bats at 17.8 hours at 22°C and 1.8 hours at 37°C [35]. The half-life in Mango flesh was 30.3 hours at 22°C and 2.2 hours at 37°C, but only 12.2 hours in lychee juice at 22°C and 1.45 minutes for desiccation at 22^o^C [35]. An experimental study on Hendra virus (a paramyxovirus closely related to Nipah virus) estimated the half-life at 4^o^C to be 308 hours [36]. To describe the change in half-life by temperature in the different media (bat urine, Mango flesh and Lychee), we fit exponential curves to these data, *y=Ae^-Bt^*, using the value of 308 hours at 4°C as a reference data point for all models to give 3 data points for each model, see Table 6. From these models we can estimate the half-lives at different temperatures; we use the bat urine model at 30°C to estimate virus reduction pre-harvesting, C*_HL_(1,j,k,l,m),* as it is the closest to summer temperatures in South East Asia, and the Mango Flesh model at 4°C to estimate virus reduction during transport, C*_HL_(3,j,k,l,m),* as it is assumed the products would be chilled during transport and so stored at similar temperature.

**Table 6: parameter estimates for NiV half-life in different media and the R^2^** model fit parameter.

| **Model** | **(A,B) parameter estimates** | **R^2^ value** |
| --- | --- | --- |
| Bat Urine | (662.33,0.156) | 0.9999 |
| Mango Flesh | (725.05,0.149) | 0.9927 |
| Lychee | (753.82,0.162) | 0.9963 |

*Reduction due to processing, V(2,j,k,l,m).* This parameter accounts for a reduction in the probability of contamination due to product specific effects such as heating, drying, fermenting, washing and peeling. As reported in a previous risk assessment [37], very little work has been done on survival of bat-borne viruses on foodstuffs. Estimates of reduction in virus concentration throughout the processing chain are therefore generally based on evidence from studies using enteric viruses known to survive the enzymatic and extreme pH conditions of the gastrointestinal tract and therefore tend to persist in the environment. Products were categorised according to whether they were raw products, products which had undergone some preparation or processing and products which had undergone treatments such as heating, sterilising or high pressure processing (Table 7). Each category was then assessed using available literature on the effectiveness of each process in reducing virus concentration and a log reduction was estimated for each category. Categories were kept as generic as possible due to a lack of data to determine a different risk at a more refined level. Thus, for example, although processed products can include dried raw products or a product which has been extracted from the raw state such as flour, they are all assumed to have similar reduction in virus concentration as a result of the different processing stages.

**Table 7**: Estimated log reduction of virus under the different processing methods

| **Description** | **Definition** | **Log reduction** | **Refer-**  **ence** |
| --- | --- | --- | --- |
| Raw Product | Live animal or part of animal which has not undergone any cooking. Crop which is exported from harvest with no preparation or processing. | 0 | [38,39] |
| Prepared Product | Includes fruit and veg which has been washed and sanitised; shelled nuts; primary products e.g. wheat germ etc. | 1 | [38,40-45] |
| Processed product | Includes, dried fruit and veg; secondary products such as pasta, starch, beer etc. | 2 | [46,47] |
| Chemically processed product | Product which has been extracted using solvents e.g. oils etc. or chemically manufactured from raw product | 3 | [48] |
| Thermal/high pressure treated product | Product which has undergone a cooking process such as boiling, baking, roasting etc. or high pressure processing | 3 | [46,49-51] |
| Thermal/high pressure treated conc. product | Includes canned products, pasteurised fruit and vegetable juices | 4 | [52-54] |

The model does not consider further contamination during processing is not dependent on the human infection prevalence, although acknowledge that there could be a small risk due to cross-contamination, direct contact with infected livestock or infected producers handling the products during preparation.

*Minimum concentration of virus for contaminated product, C_min._* Experimental infection of hamsters and ferrets suggests that a dose of 500 TCID_50_ is not enough to guarantee infection [55] and an experimental study on African green monkeys suggested doses of at least 2.5*10^3^ pfu/ml were sufficient to cause infection [56]. Thus we assume that *C_min_*= 1 log_10_ TCID_50_/g, as a worst case scenario.

*Duration of journey between exporting country and EU MS, T_HL_(3,j,k,l,m).* The journey duration *T_DM_(j,k,m)* between the exporting country, *k*, and the MS, *j*, is estimated by dividing the great circle distance (miles) between the two countries by the average speed of transport (which we assume to be 500 mph for an aircraft and 25mph for sea travel). This is a simplification of the actual duration as it uses an average longitude and latitude within the countries (as given in the *wrld_simpl* map from the *maptools* R library) rather than the location of the actual ports (which are unknown in the dataset used). For big countries, such as India, Russia and China, this could lead to a considerable difference in the estimated, versus actual duration. Further, this calculation assumes that the flights are direct between the countries with no stopovers, which is unlikely for long distance flights where changes are often necessary. However, this great circle distance method provides a reasonable approximation for this model.

**Live animals**

*Prevalence of infection in other animals, p_Ainf_(k).* There is no good data for specific parameterisation of NiV in other animals. Thus, we assume that the prevalence is going to be more similar to that in humans than that in bats, as bats are the reservoir host species and must pass it on to other animals, which they are assumed to do so at a similar rate as to humans. Thus, in the absence of better data, we estimate the prevalence in live animals based on the human infection prevalence, *p_Hinf_(k)*, by assuming it is equivalent to the mean prevalence in the human population over all exporting countries with human infection, (so that we do not equate absence of human infection with absence in animal population).

*Number of animals, N_A_(j,k,s)*.

Data are available from the TRACES database on the number of animals that are brought into the EU and issued with a CVEDA permit [57]. These data provide the country of import and export along with the quantity of animals transported broken down by commodity codes. However, many of the species we might be interested in, such as cats and dogs, are recorded under the ‘other’ code. We therefore defined our own extra species codes to account for each species independently within the model, which we applied to the data.

The number of permits by exporting country *j* and MS *k,* of each species/code between 2011-2013 were combined, *N_A1_(s,j,k)*. A single permit may apply to multiple animals. Thus, the average number of animals per permit per species, *N_A2_(s),* is calculated by

$N_{A2}(s)=\sum_{j_{a}(s)=1}^{J_{a}(s)} j_{a}(s)*p_{j}\left(j_{z}(s)\right)$,

where *j_a_(s)* is the number of animals per permit, *J_a_(s)* is the maximum number of animals per permit and *p_j_(j_a_(s))* is the proportion of all permits of species *s* with *j_a_(s)* animals. Note that *N_A2_(s)* is not country specific, as it was considered that there was insufficient data at this level for parameterisation. Thus, the estimated total number of non-food animals transported from exporting country *j* to MS *k*, *N_A_(s,j,k)*, is given by

$N_{A}(s,j,k)=N_{A1}(s,j,k)*N_{A2}(s)$.

In using this dataset, there is ambiguity in defining the MS, *j*: The TRACES data indicates both the country of destination and the border inspection post where the check was carried out. These are often not the same country and the country of destination is not always in the EU. It was decided that pets with a destination outside the EU should still be considered, as they will have stopped off in the EU to undergo inspection and thus have entered the EU.

*Species of animal, s.* The following species were considered as susceptible to NiV due to previous research: , all non-human Primates, *Canis familiaris (dog), Felis Catus (cat)* and *Mustela putorius furio (ferret), Sus scrofa domesticus (pig)* [6,56,58,59]. Data on imports of these species were obtained from TRACES.

**Illegal bushmeat**

*Number of bushmeat seizures, N_seized_(i,j,k).* Unpublished data are used on the number of seizures of bushmeat from passengers at UK airports by country of origin. However, these data are limited and so we aggregate by Sub Region of the world, *k^*^,* as defined by the United Nations (<http://unstats.un.org/unsd/methods/m49/m49regin.htm>).

Additionally, there are no denominator data included in the dataset so the total number of consignments of illegal bushmeat entering the UK or the total number of luggage searched is unknown.

We also assume that only passengers resident in country *k* and any passengers who have been visiting friends or relatives could bring back bushmeat

$$\begin{matrix} N_{seized}(i,j,k)=\left\{\begin{matrix} N_{seized}(i,j,k^{*}) & k\in k^{*},i\in\{FB,FH,FVFR,MSVFR,MISC,CON\} & \\ 0 & i\in\{MSH,MSB\} & \end{matrix}\right. \\ \end{matrix}$$

*Under-reporting factor, p_UF._* A study on illegal meat entering Switzerland used a model to derive estimates for the annual proportion of searched luggage [60]. They estimated this proportion to be 0.24% (95% CI 0.15 to 0.43) at Zurich airport and 0.06% (95% CI 0.03 to 0.2) at Geneva airport. These model estimates were lower than the expert opinion estimates of people working at the airports (0.5% in Zurich and 0.25%-0.44% in Geneva).

With targeted testing of passengers for illegal meat products it is likely that the proportion of people smuggling bushmeat who have their luggage searched is actually higher than the proportion of the total population. For example, assume that 10% of people on average have their luggage searched, then if 10 people had their luggage searched we would expect there to be 100 passengers. However, people smuggling bushmeat might be more likely to be stopped, so if 10 people were found to have bushmeat then we might expect the total number of people smuggling bushmeat to be less than 100 people.

Thus, based on these data, we use the larger estimate of searched luggage and assume that *p_UF_*= 0.5% of people entering airports will have their luggage searched.

*Number of passengers travelling to MS j, N_H_(i,j,k).* As the data used to estimate *N_seized_(i,j,k)* are from the UK, it is only appropriate to use the UK information on number of passengers, as discussed previously these data are obtained from Eurostat; *N_H_(i,j,k), for j=UK*.

*Species of bushmeat, p_BMSp_(s).* Previous published studies on bushmeat in Europe have not identified bats among seizures of bushmeat sampled [60,61]. However there are reports of bat busmeat being sold in the EU, although not *Pteropus* *spp.* [62]. A study reported on the species of bushmeat items confiscated at US ports of entry between 2005-2010, suggested that bats accounted for around 1.5% of all bushmeat [63]. Thus, in the absence of other information, we assume that 1.5% of bushmeat is bats.

*Probability bushmeat is contaminated, p_BMcontam_(s,k)*. We assume that the probability a consignment of bushmeat is contaminated depends on the species of animal the bushmeat came from. If the species is a bat then the probability of contamination is equal to the prevalence of infection in the bat population in that country, *p_BInf_(k),* otherwise it is equivalent to our estimate for prevalence in live animals, *p_Ainf_(k).*

**References**

1. **Passengers on board**: All passengers on board of the aircraft upon landing at the reporting airport or at taking off from the reporting airport. All revenue and non revenue passengers on board an aircraft during a flight stage. Includes direct transit passengers (counted at arrivals and departures).

   **Passengers Carried**: All passengers on a particular flight (with one flight number) counted once only and not repeatedly on each individual stage of that flight. All revenue and non-revenue passengers whose journey begins or terminates at the reporting airport and transfer passengers joining or leaving the flight at the reporting airport. Excludes direct transit passengers. <http://epp.eurostat.ec.europa.eu/cache/ITY_SDDS/EN/avia_pa_esms.htm> [↑](#footnote-ref-2)
